# Targeting the PI3K/mTOR Pathway in Pediatric Hematologic Malignancies

**DOI:** 10.3389/fonc.2014.00108

**Published:** 2014-05-16

**Authors:** Sarah K. Tasian, David T. Teachey, Susan R. Rheingold

**Affiliations:** ^1^Division of Oncology, Department of Pediatrics, The Children’s Hospital of Philadelphia, University of Pennsylvania School of Medicine, Philadelphia, PA, USA

**Keywords:** acute lymphoblastic leukemia, acute myeloid leukemia, clinical trial, Hodgkin lymphoma, non-Hodgkin lymphoma, pediatric, PI3K/mTOR, tyrosine kinase inhibitors

## Abstract

A complex interplay of intracellular signaling networks orchestrates normal cell growth and survival, including translation, transcription, proliferation, and cell cycle progression. Dysregulation of such signals occurs commonly in many malignancies, thereby giving the cancer cell a survival advantage, but also providing possible targets for therapeutic intervention. Activation of the phosphatidylinositol 3-kinase (PI3K)/protein kinase B (Akt)/mammalian target of rapamycin (mTOR) signaling pathway contributes to the proliferative advantage of malignant cells and may confer resistance to chemotherapy in various hematologic malignancies. The initial mTOR inhibitor, sirolimus (also known as rapamycin), was first discovered in 1975 in the soil of Easter Island. Sirolimus was originally developed as an anti-fungal agent given its macrolide properties, but was approved by the Food and Drug Administration (FDA) in 1999 as an immunosuppressive agent for renal transplantation patients once its T cell suppression characteristics were recognized. Shortly thereafter, recognition of sirolimus’s ability to inhibit cellular proliferation and cell cycle progression brought sirolimus to the forefront as a possible inhibitor of mTOR. In the subsequent decade, the functional roles of the mTOR protein have been more fully elucidated, and this protein is now known to be a key regulator in a highly complex signaling pathway that controls cell growth, proliferation, metabolism, and apoptosis. This article discusses the dysregulation of PI3K/mTOR signaling in hematologic malignancies, including acute and chronic leukemias, lymphomas, and lymphoproliferative disorders. The current repertoire of PI3K/mTOR pathway inhibitors in development and clinical trials to date are described with emphasis upon pediatric hematologic malignancies (Figure 1). Investigation of small molecule inhibitors of this complex signaling network is an active area of oncology drug development.

## The PI3K/mTOR Signaling Network

The PI3K/mammalian target of rapamycin (mTOR) pathway is involved in many critical cellular functions, including protein synthesis, cell cycle progression, apoptosis, and drug resistance. Growth factors, such as insulin-like growth factor (IGF), interleukin 7 (IL-7), and fms-like tyrosine kinase 3 ligand (FLT3-L), bind their tyrosine kinase receptors on the cell surface membrane and induce intracellular PI3K/mTOR pathway signaling. Activation of PI3K recruits cellular protein kinases that in turn activate downstream kinases, including the serine/threonine kinase Akt. Phosphorylation of Akt at its threonine 308 residue inactivates the tuberous sclerosis complexes 1 and 2 (TSC1 and TSC2), permitting activation of the mTOR complex 1 (mTORC1) and subsequent phosphorylation of proteins 70S6K1, S6, and the eukaryotic translation initiation factor 4E-binding protein 1 (4EBP1). This activation of mTORC1 results in increased translation and protein synthesis. A second complex of mTOR, known as mTORC2, has been more recently described and appears to act as a feedback loop via Akt. Activation of mTORC2 via phosphorylated 70S6K1 induces phosphorylation of Akt at its serine 473 residue, thereby inhibiting mTORC1 activation and resulting in G1 cell cycle arrest and apoptosis via 4EBP1. The tumor suppressor protein PTEN (phosphatase and tensin homolog deleted on chromosome 10) provides additional negative regulation of the PI3K/mTOR pathway via inhibition of Akt and phosphatidylinositol (3,4,5)-triphosphate (PIP_3_); loss of PTEN results in upregulation of PI3K-mediated signaling (1–5).

Oncogenesis may result from various mechanisms that disrupt the PI3K/mTOR pathway. Increased surface expression of growth factor receptors is one mechanism, such as overexpression of the IGF receptor in some leukemias (6). Mutation of intracellular downstream effectors, including PI3K/mTOR pathway proteins, may also result in constitutive activation of signaling. Loss of tumor suppressors, such as PTEN, can induce dysregulation of normal cellular equilibrium and thereby facilitate aberrant signaling activation (7).

## Preclinical Data in Pediatric Hematologic Malignancies

### Acute lymphoblastic leukemia

Modern genomic techniques and biochemical assays have been used to identify upregulation of the PI3K/mTOR pathway in both pediatric B and T-acute lymphoblastic leukemia (ALL) (3). Various abnormalities within the PI3K/mTOR pathway have been identified in different immunophenotypic and molecular subsets of pediatric ALL, which may help to guide the selection of new agents for preclinical and clinical testing. Numerous strategies have been used to target aberrant PI3K/mTOR pathway signaling in leukemias (1–3, 8, 9). To date, small molecule inhibitors of the mTOR protein (mTOR inhibitors; MTIs) have been best investigated in the preclinical and clinical arenas. This class of drugs includes the first MTI, sirolimus, as well as structural derivatives or analogs (“rapalogs”) such as temsirolimus, everolimus, and ridaforolimus. Initial studies revealed that MTIs inhibit growth and induce apoptosis in ALL cell lines and primary ALL cells *in vitro*, as well as inhibit leukemia proliferation *in vivo* in human ALL xenograft models (10, 11). Testing by the pediatric preclinical testing program (PPTP) demonstrated significant leukemia cytotoxicity in five of eight ALL cell lines treated with sirolimus, particularly in T-ALL cell lines (12). *In vivo* testing of sirolimus in immunocompromised murine xenograft models of pediatric ALL through the PPTP resulted in significant survival differences in five of eight sirolimus-treated animal models in comparison to vehicle controls (12).

mTOR inhibitors have been found to have additive or synergistic effects when combined with cytotoxic chemotherapy agents, as well as to reverse chemotherapeutic resistance in some leukemias. Wei et al. demonstrated resensitization of lymphoid malignancy cells to glucocorticoid-induced apoptosis when cells were first treated with sirolimus and that these effects were modulated at least in part by the anti-apoptosis protein Mcl-1 (13). Further assessment by the PPTP of combination sirolimus and dexamethasone demonstrated superior *in vitro* cytotoxicity in six of seven ALL cell lines and *in vivo* efficacy in one of three ALL xenograft models in comparison to single-agent treatment (14). However, combination of sirolimus with other cytotoxic agents was not additive in these studies. Teachey et al. showed that MTIs had at least an additive or synergistic effect *in vitro* upon ALL cell lines when combined with methotrexate, dexamethasone, l-asparaginase, etoposide, or doxorubicin (15). MTIs also were found to downregulate cyclin D1, leading to decreased dihydrofolate reductase (DHFR) synthesis and possibly increased sensitivity of ALL blasts to methotrexate (15). Combination of the MTI temsirolimus with methotrexate resulted in long-term survival of some human B-ALL xenograft models. Crazzolara et al. noted increased survival of B-ALL xenograft mice treated with the MTI everolimus and vincristine in comparison to mice treated with vincristine or everolimus alone (16). Many studies suggest that MTIs may be more active in ALL types with worse outcomes, including T-ALL, *BCR-ABL1*-rearranged, and *BCR-ABL1*-like (“Philadelphia-like”) B-ALL (11, 17–20).

### BCR-ABL1-positive and BCR-ABL1-like B-ALL

The BCR-ABL1 fusion protein resulting from *t*(9;22) activates the PI3K/mTOR pathway directly in both chronic myeloid leukemia (CML) and Philadelphia chromosome-positive (Ph+) ALL (17). Although tyrosine kinase inhibitors (TKIs) targeting mutant ABL1 have revolutionized treatment of Ph+ leukemias in adults and children, resistance mutations are a known sequelae of TKI therapy that often result in eventual treatment failure. One study recently demonstrated improved growth inhibition and induction of apoptosis when sirolimus was added to imatinib-resistant Ph+ ALL cell lines harboring an acquired T315I resistance mutation (18). BCR-ABL directly activates the PI3K/mTOR pathway, which may explain some of the robust preclinical activity of MTIs in these leukemias (17).

Recent genomic profiling efforts have identified the Philadelphia chromosome-like (Ph-like) subtype of B-ALL, which has a kinase-activated gene expression profile similar to that of *BCR-ABL1-*rearranged ALL and is associated with various genetic alterations known or predicted to activate oncogenic cytokine receptor signaling (21–23). Adults and children with Ph-like ALL respond poorly to cytotoxic chemotherapy and experience high rates of relapse. Approximately half of Ph-like ALLs harbor mutations in Janus kinase (JAK) pathway proteins and/or rearrangements in the cytokine receptor-like factor 2 gene (*CRLF2*). The CRLF2 protein heterodimerizes with the IL-7 receptor alpha chain to form the thymic stromal lymphopoietin receptor (TSLPR) complex. Initial studies using (non-*CRLF2*-rearranged) murine B-ALL models identified activation of PI3K/mTOR signaling in these leukemias, which was modulated by IL-7 and TSLP and could be abrogated *in vitro* and *in vivo* by sirolimus treatment (24, 25). Subsequently, constitutive activation of both JAK/STAT and PI3K/mTOR signaling was reported specifically in *in vitro* analyses of primary pediatric ALL specimens with JAK mutations and/or *CRLF2* rearrangements (19). Aberrant signaling was abrogated *in vitro* with co-incubation of ALL cells with TKIs, including the JAK inhibitor ruxolitinib and various inhibitors of the PI3K/mTOR pathway (19). The efficacy of mTOR (and JAK) inhibition has been further studied *in vivo* in pediatric Ph-like ALL xenograft models. Sirolimus treatment significantly inhibited leukemia proliferation in eight tested models with JAK pathway mutations and/or *CRLF2* rearrangements, as well as resulted in enhanced long-term animal survival (26). Other PI3K/mTOR pathway-targeting approaches using PI3K isoform-selective or dual protein inhibitors have also demonstrated preliminary efficacy *in vivo* using these xenograft models (20). The therapeutic potential of PI3K/mTOR inhibition in B-ALL remains under active preclinical investigation.

### T-ALL

Aberrant Notch-1 receptor signaling occurs commonly in T-ALL, and approximately half of T-ALL patients have somatic *NOTCH1* mutations (27, 28). The intracellular Notch-1 protein, when released after cleavage by gamma secretase, has a direct effect on gene expression, but it has also been found to upregulate PI3K/mTOR pathway signaling via degradation of PTEN (27). In T-ALL, inactivation of PTEN leads to upregulation of Akt and mTOR, in turn leading to upregulation of cell growth and proliferation (7, 28). PTEN is inactivated through a variety of mechanisms including mutation or deletion of *PTEN* itself or defects in other signaling pathways (e.g., Ras), leading to decreased transcription or modification of PTEN (29).

Gutierrez et al. performed array CGH and sequencing on DNA from 44 pediatric T-ALL specimens to determine the incidence of PI3K pathway mutations (7). They found 4 patients with *PTEN* deletions, 12 cases with novel *PTEN* exon 7 mutations, 1 *AKT* mutation, and 4 mutations involving genes encoding PI3K proteins. Alterations were mutually exclusive and in total occurred in 47.7% (21 of 44) of pediatric T-ALL cases assessed. Correlation of mutation status with clinical outcomes data revealed that T-ALL patients with complete deletions of *PTEN* were far more likely to experience induction failure (>25% residual leukemia after 1 month of chemotherapy) than those with mutated *PTEN* (7). Gain-of-function mutations in genes encoding the IL-7 receptor, a previously identified MTI-sensitive target, have also been reported in T-ALL (30, 31).

T-ALL cell lines and primary T-ALL samples with alterations in the PI3K/mTOR pathway have been analyzed for their therapeutic potential in the laboratory. MTIs as single-agents *in vitro* have proven mainly cytostatic in leukemias, likely due to feedback loops that hyperactivate Akt in response to mTORC1 downregulation. Nonetheless, the *in vivo* responses to rapalogs using murine models of T-ALL seem to be superior to B-ALL. As described above, MTIs in combination with chemotherapy or as dual PI3K pathway protein inhibition approaches may more successfully induce leukemia cytotoxicity and apoptosis (15, 28).

### Acute myeloid leukemia

Constitutive activation of PI3K/mTOR signaling occurs in the majority of human acute myeloid leukemia (AML) and likely results from a variety of mechanisms, including alterations in cellular growth factors and mutations in FLT3, c-kit, and Ras pathway proteins (32–34). Various MTIs appear to be cytostatic or cytotoxic when incubated with primary AML samples *in vitro* and have proven synergistic with AML-directed cytotoxic chemotherapy in *in vivo* AML mouse models (35–37). Use of “next-generation” inhibitors that more fully inhibit mTORC1 and 4EBP1 phosphorylation are of current interest in AML and are under active preclinical investigation (2, 38). Promising preclinical data also support use of MTIs in combination with conventional chemotherapy or with histone deacetylase (HDAC) inhibitors, which have led to several combination therapy clinical trials in adults (33). As aforementioned, PI3K/mTOR signaling is hyperactive in CML, and investigation of potential synergy between MTIs and TKIs targeting mutant ABL1 (e.g., imatinib, dasatinib) in CML is planned (NCT01188889) (39). Such combination approaches may be particularly useful for overcoming of TKI resistance in CML with acquired mutations (40, 41).

### Myeloproliferative neoplasms

Many myeloproliferative neoplasms (MPNs) harbor point mutations in *JAK2* (Janus kinase 2) that result in signaling hyperactivation involving signal transduction and activator of transcription factors (STATs), but constitutive PI3K/mTOR activation has also been implicated in MPNs. Everolimus was shown to inhibit cell proliferation of *JAK2* V617F mutant cell lines, and the dual mTORC1/2 inhibitor PP242 even more potently induced apoptosis (42). Further, combination of the JAK1/2 inhibitor ruxolitinib and everolimus resulted in synergistic inhibition of cell proliferation and cell cycle arrest in MPN models *in vitro* and *in vivo* (43).

### Non-Hodgkin lymphoma

The majority of B-cell lymphomas also have been found to have constitutive activation of the PI3K/mTOR pathway, and MTIs have shown anti-lymphoma activity both *in vivo* and *in vitro* (44, 45). The mechanism by which the pathway is activated differs by lymphoma subtype. Cyclin D1 is overexpressed in mantle cell lymphoma, a cell cycle protein downstream of Akt and sensitive to mTOR inhibition (44–46). PI3K appears to be constitutively activated in Burkitt lymphoma cell lines (47). In a mouse model, *CMYC*, a hallmark translocated gene in Burkitt lymphomas, appears to cooperate with PI3K to create the oncogenic phenotype (47). Co-culture of Burkitt cell lines with various PI3K inhibitors resulted in decreased phosphorylation of Akt and 70S6K1 and subsequent cell death (48).

Abnormal activation of 70S6K1 has been described in over 90% of adult diffuse large B-cell lymphoma (DLBCL) cases, but is less well-defined in pediatric DLBCL (49, 50). Preclinical testing of Burkitt lymphoma and DLBCL cell lines with MTI monotherapy or MTIs in combination with HDAC inhibitors shows enhanced cell apoptosis versus single-agent HDAC and substantial inhibition of cell cycle progression, primarily due to cell cycle arrest at G0/G1 (49–51). This improved efficacy has been attributed to HDAC inhibitors’ ability to block compensatory mTORC2-mediated Akt upregulation that results from MTI effects upon mTORC1 (51).

Akt is frequently constitutively activated in ALCL harboring *ALK* (anaplastic lymphoma kinase) mutations, and targeting with MTIs in preclinical models has shown some activity (52).

### Hodgkin lymphoma

Genomic interrogation of Hodgkin lymphoma cell lines has identified dysregulation of the PI3K/mTOR pathway and high AKT phosphorylation (53, 54). *In vitro*, treatment with an isoform-selective PI3K inhibitor alone and in combination with MTIs inhibited Hodgkin cell line proliferation (55). The combination of the MTI everolimus and the HDAC inhibitor panobinostat has also demonstrated synergy in induction of cell death in preclinical testing (56).

### Lymphoproliferative disorders

Dysregulated mTOR signaling occurs in a variety of lymphoproliferative disorders, including autoimmune lymphoproliferative syndrome (ALPS), a disorder of lymphocyte survival due to defective apoptosis hallmarked biologically by double negative T-cells (1). mTOR has also been shown to be constitutively activated in post-transplant lymphoproliferative disease (PTLD) and viral-induced lymphoproliferative disease in children with immunodeficiencies. First line intervention is often reduction of immunosuppression at the cost of graft rejection, but sirolimus offers both T-cell suppression and activity against the lymphoproliferation (57, 58).

## Clinical Trials of PI3k/mTOR Pathway Inhibitors

### Pediatric clinical trials of mTOR inhibitors in hematologic malignancies

An institutional Phase 1 trial tested the safety of oral sirolimus monotherapy in nine pediatric patients with relapsed ALL. No dose limiting toxicity (DLT) was noted, and three ALL patients met the criteria for stable disease (59). Based upon these data, a subsequent single institution study is currently testing sirolimus in combination with methotrexate in relapsed/refractory pediatric ALL (NCT01162551) (39). In another institutional trial, five pediatric and adult patients with relapsed ALL were treated with a combination of sirolimus and prednisone for 5 days prior to starting multi-agent re-induction chemotherapy (60). Changes in gene expression and protein levels, including Mcl-1, were measured in leukemia samples from these patients and compared with samples from ALL patients treated with glucocorticoids alone. Several patients had evidence of decreased Mcl-1 protein levels and inhibition of phosphorylated S6 (pS6) after initiation of therapy, suggesting successful mTOR inhibition. Among patients treated with corticosteroids alone, most demonstrated no change in Mcl-1 or pS6 levels. All patients treated with sirolimus and prednisone demonstrated a decrease in circulating blasts (>80% decrease from baseline) during the 5 days window (60). A successor trial of oral everolimus in combination with maintenance-like ALL therapy is currently enrolling (NCT01523977) (39).

A multi-institutional Children’s Oncology Group (COG) Phase 1 trial combined 3-weekly IV temsirolimus (10 mg/m^2^, de-escalated to 7.5 mg/m^2^) with an intensive four drug re-induction backbone per the UKALL R3 relapse protocol (NCT01403415) (39, 61). Due to significant infectious toxicity, the trial was suspended and recently reopened with two doses of temsirolimus at 7.5 mg/m^2^. A companion trial will soon be open through the Therapeutic Advances in Childhood Leukemia Consortium combining two doses of IV temsirolimus with the standard 5 days ALL salvage therapy of etoposide and cyclophosphamide. A single institution trial with oral sirolimus on the UKALL R3 backbone is also currently open (NCT01658007) (39).

Sirolimus treatment has also been studied in a limited institutional series in children with ALPS. These patients have traditionally been treated with immunosuppression such as steroids and solid organ transplant rejection/prophylaxis medications. Five out of five patients with steroid-refractory ALPS had complete responses (CRs) with resolution of lymphadenopathy with single-agent (1). An ongoing trial for pediatric patients with refractory ALPS has treated 12 patients to date, and 10 of these children had CRs with resolution of lymphadenopathy and normalization of blood counts (NCT00392951) (39). Eight of these patients also eradicated their double negative T-cell population, a hallmark of this disease (62). Children with other refractory autoimmune-induced cytopenias (e.g., resulting from systemic lupus erythematosus or Evans syndrome) have also experienced clinical responses with sirolimus monotherapy (62).

### Adult clinical trials of mTOR inhibitors in hematologic malignancies

A Phase 1/2 study of oral everolimus in adult patients with relapsed hematologic malignancies was recently reported (63). No DLTs were noted, although Grade 3 toxicities occurring in more than 5% of patients included hyperglycemia (22%), hypophosphatemia (7%), and fatigue (7%). The only published combination trial of an MTI and high-dose chemotherapy in AML is an adult Phase 1 dose escalation trial of oral daily sirolimus on a backbone of standard adult AML salvage therapy consisting of 5 days each of mitoxantrone, cytarabine, and etoposide (MEC). No increased toxicity with the MTI/chemotherapy combination occurred, and responses (complete and partial) occurred in 22% of patients. One patient treated at the highest dose level experienced a DLT of prolonged marrow suppression. Overall, sirolimus was well tolerated and did not appear to increase non-hematologic toxicity related to multi-agent chemotherapy (64).

A Phase 1 trial of oral everolimus in combination with cytarabine in elderly adults with *de novo* AML showed improved median overall survival when compared to cytarabine alone (65). Everolimus in combination with 7 + 3 cytarabine and daunomycin in refractory/relapsed AML was fairly well tolerated and had a CR rate of 68%, showing some improvement over historical controls (66). A single-agent everolimus Phase 1/2 trial in adults with myelofibrosis showed a 23% response rate with over 80% of the patients experiencing symptomatic benefit (67).

Two Phase 2 studies of single-agent temsirolimus in adults with relapsed or refractory mantle cell lymphoma have been conducted to date (68, 69). In one study, objective responses were seen in 13 of 34 patients [one CR and 12 partial responses (PRs)]. The median time-to-progression in all patients was 6.5 months, and the median duration of response for the responders was 6.9 months (44). In the second study, 29 patients were treated and responses were seen in 41% of patients (1 CR and 10 PRs) (45). Phase 2 trials of single-agent everolimus in patients with refractory Hodgkin lymphoma (HL) revealed a 42–47% overall response rate (53). Based upon preclinical data showing synergy with combined mTOR and HDAC targeting, a Phase 1 trial in both relapsed non-Hodgkin lymphoma (NHL) and HL of everolimus and the HDAC inhibitor panobinostat was performed and resulted in a similar 43% response rate in patients with HL. Some responses were also observed in NHL patients (70).

### Adult clinical trials of “next-generation” PI3K/mTOR pathway inhibitors in hematologic malignancies

Advances in structural and biochemical understanding of PI3K enzymes have facilitated the development of new inhibitors targeting other signaling proteins of the PI3K/mTOR pathway. Many of these agents have favorable pharmacologic properties and have proven highly efficacious in preclinical cancer studies, thus generating significant interest for clinical development of promising inhibitors. In particular, many of these drugs are predicted or known to induce cytotoxicity in comparison to the generally cytostatic effects of earlier MTIs (2, 9, 71). However, the clinical efficacy against human cancers and the side effect profiles of these drugs upon normal tissues remain to be characterized fully (38).

**Figure 1:**
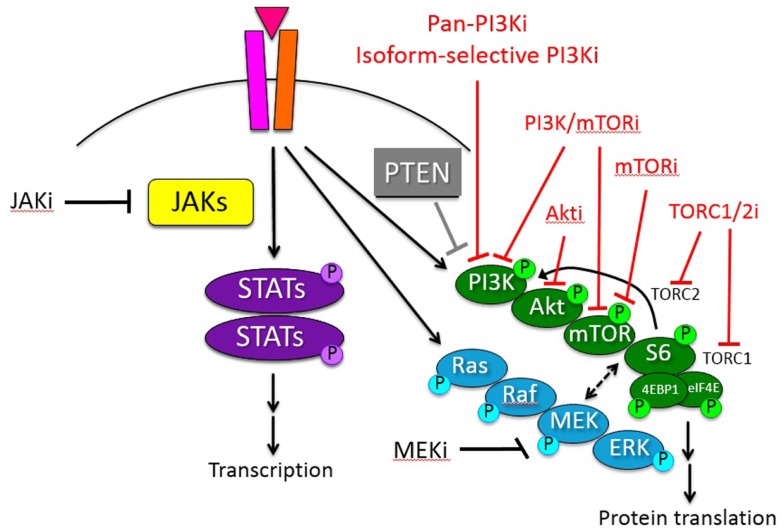
**Schema of PI3K/mTOR and other oncogenic signaling pathways in hematologic malignancies**. Inhibitors (i) of these signaling proteins under current preclinical or clinical study with potential therapeutic relevance for pediatric hematologic malignancies are delineated. Red = inhibitors of PI3K/mTOR pathway signal transduction protein(s).

Currently, six classes of PI3K/mTOR pathway inhibitors have been developed: (a) pan-class I PI3K inhibitors, (b) isoform-selective PI3K inhibitors, (c) Akt inhibitors, (d) MTIs/rapalogs, (e) dual PI3K/mTOR inhibitors, and (f) TORC1/TORC2 inhibitors (also known as TORKinibs or active-site mTOR inhibitors) (Figure 1). All of these agents remain under active preclinical study, and combination approaches of PI3K/mTOR pathway inhibitors and other signal transduction inhibitors in hematologic malignancies are also in progress in the laboratory (43, 72, 73). Many of the newer non-MTI drugs have been evaluated clinically in the phase 1/2 setting in adults with relapsed/refractory solid tumors. A smaller number of mainly early phase trials evaluating next-generation PI3K/mTOR pathway inhibitors in adults with relapsed/refractory hematologic malignancies are currently open. Particular progress has been made in studying the pan-PI3K and isoform-selective PI3K inhibitors in patients with advanced leukemias and lymphomas, including recent FDA approval of the PI3K p110δ isoform-selective inhibitor idelalisib for indolent NHL (39, 71). To date, none of the next-generation PI3K/mTOR pathway inhibitors has been evaluated specifically in children, although preclinical studies of many of these drugs are ongoing in childhood leukemias with the goal of swift clinical translation (20, 74).

## Toxicity

mTOR inhibitors are generally well tolerated as single-agents with common side effects such as hyperglycemia, hyperlipidemia, hepatic impairment, infection, fatigue, and mucositis (44). These toxicities have been found to be generally manageable in patients following solid organ transplantation and in single-agent oncology trials. Pancytopenia, most notably thrombocytopenia, has been noted in solid tumor and NHL trials (44, 46, 51, 75). However, MTIs have also been found to potentiate the toxicities of cytotoxic chemotherapies, especially drugs known to cause mucositis, poor wound healing, hypertension, and hyperglycemia (44). Although sirolimus was not associated with increased toxicity when administered with multi-agent chemotherapy in adults with AML in one pilot study (64), early phase clinical trial experience with temsirolimus in combination with anti-ALL chemotherapy in children resulted in unacceptable toxicity and required dose de-escalation (SR Rheingold, personal communication 2014). Clinical evaluation of next-generation PI3K/mTOR pathway inhibitors (Table 1) remains at an early stage, but toxicities appear to be related to immunosuppression and associated infectious sequelae, effects upon cellular metabolism, gastrointestinal symptoms, and skin rash (8, 38, 71). Significant toxicities may preclude further clinical development of some of these next-generation inhibitors, but efforts are ongoing to identify efficacious PI3K/mTOR pathway inhibitors with tolerable side effect profiles (8, 38, 71). In addition, the safety of most of these agents in children with cancer remains to be established.

**Table 1:** **PI3K/Akt/mTOR inhibitors in clinical trials for hematologic malignancies**.

	Target(s)	Phase of testing	Clinical trial number
**MTOR INHIBITORS**			
Sirolimus (rapamycin)	mTOR	FDA-approved	*NCT01162551 *NCT01658007 *NCT01670175
Temsirolimus (CCI-779)	mTOR	FDA-approved	*NCT01403415 (COG ADVL1114) *NCT01614197 (TACL 2008-004)
Everolimus (RAD001)	mTOR	FDA-approved	*NCT01523977
Ridaforolimus (AP23573)	mTOR	FDA-approved	completed
**PI3K INHIBITORS**			
BKM120	Pan-PI3K (class I)	Phase 1/2	NCT01396499 NCT01660451 NCT01693614 NCT01719250 NCT02049541
BAY80-6946	Pan-PI3K (class I)	Phase 1/2	NCT01660451
BYL719	PI3K p110α	Phase 1/2	NCT01905813
GSK2636771	PI3K p110β	Phase 1/2	NCT01458067
SAR260301	PI3K p110β	Phase 1	NCT01596270
Idelalisib (GS-1101, CAL-101)	PI3K p110δ	FDA-approved	Multiple single-agent and combination trials
INCB040093	PI3K p110δ	Phase 1	NCT01905813
AMG 319	PI3K p110δ	Phase 1	NCT01300026
TGR 1202	PI3K p110δ	Phase 1	NCT01767766
IPI-145	PI3K p110γ/δ	Phase 1/2/3	NCT02004522 NCT01882803 NCT01871675 NCT01476657
**PI3K/MTOR INHIBITORS**			
BEZ235	PI3K/mTOR	Phase 1/2	NCT01756118
SAR245409	PI3K/mTOR	Phase 1/2	NCT01410513 NCT01403636
GDC-0980	PI3K/mTOR	Phase 1/2	NCT00854152
VS-5584	PI3K/mTOR	Phase 1	NCT01991938
**AKT INHIBITORS**			
MK-2206	Akt	Phase 1/2	NCT01369849
GSK2110183	Akt	Phase 1/2	NCT00881946 NCT01532700
**MTORC1/MTORC2 INHIBITORS**			
OSI-027	TORC1/TORC2	Phase 1	NCT00698243
DS-3078a	TORC1/TORC2	Phase 1	NCT01588678
CC-223	TORC1/TORC2	Phase 1	NCT01177397 NCT02031419
**OTHER INHIBITORS**			
CC-115	dual DNA-PK/mTOR	Phase 1	NCT01353625
CUDC-907	PI3K & HDAC	Phase 1	NCT01742988

Akt, A serine/threonine protein kinase B; COG, Children’s Oncology Group; DNA-PK, DNA-dependent protein kinase; HDAC, histone deacetylase;

mTOR, mammalian target of rapamycin; PI3K, phosphatidylinositol 3-kinase; TACL, Therapeutic Advances in Childhood Leukemia consortium

NCT, ClinicalTrials.gov identifier

*Open pediatric trials*.

## Future Directions: Rationally Designed Combinations of Multi-Pathway Inhibitors

Single-agents MTIs have shown some activity in slowing malignant cell proliferation, but are unlikely to succeed as monotherapy, as mTOR inhibition alone is often cytostatic and/or leads to compensatory upregulation of alternate signaling pathways. Identification of mTORC2 helped elucidate the path of compensatory aberrant phosphorylation noted in early MTI trials. Dual TORC1/TORC2 inhibitors have shown some improved cytotoxicity in preclinical leukemia models, but have not yet been evaluated clinically in patients with hematologic malignancies (2). As above, isoform-selective PI3K inhibitors and dual PI3K/mTOR inhibitors are under early phase clinical investigation in adults with refractory cancers based upon promising preclinical data (38, 71). Akt inhibitors under development have also shown preclinical activity in ALL with some evidence for synergy in combination with commonly used chemotherapy agents, but have notable toxicity (38, 71). Table 1 lists some of the PI3K/mTOR pathway inhibitors under evaluation in current adult clinical trials (39). Targeting of multiple PI3K/mTOR pathway proteins may lead to improved tumor suppression and/or decrease drug resistance, but may also have increased cellular toxicity. Encouraging results have also been reported in preclinical studies combining MTIs with inhibitors of complementary signaling and protein regulation pathways, including NOTCH, JAK/STAT, MYC, RAS, proteasomes, and HDACs (8, 43, 51).

Compensatory signaling and feedback loops appear to differ among leukemia and lymphoma subtypes. While preclinical results have informed the design of clinical trials to test inhibitors of the PI3K/mTOR pathway in adults and children with hematologic malignancies, significant progress remains to be made (8, 38). Toxicity due to impaired normal protein synthesis and cellular proliferation induced by these inhibitors must be better understood. The results from early phase clinical trials of combination therapy are eagerly awaited. Whether classic MTIs survive or will be surpassed by next-generation PI3K/mTOR pathway inhibitors remains to be seen. Nonetheless, MTIs remain the farthest along in clinical development and have greatly expanded our knowledge of signaling pathways essential for cell survival and proliferation.

## Conclusion

The identification of sirolimus in the soil of Easter Island has led to better understanding of its critical protein target. In the past decade, a more comprehensive description of this key signaling network required for cellular proliferation and survival has evolved. The consistent demonstration of oncogenic abnormalities in the PI3K/mTOR signaling pathway and in cross-talk pathways demonstrates a clear rationale for development of signal transduction inhibitor-based approaches for children with hematologic malignancies. Studies are ongoing to identify the most optimal inhibitor(s) for each disease subtype. Potential synergy with other targeted inhibitors and/or with conventional chemotherapy may provide additional therapeutic options to optimize therapeutic efficacy and to minimize toxicity.

## Conflict of Interest Statement

The authors declare that the research was conducted in the absence of any commercial or financial relationships that could be construed as a potential conflict of interest.
